# Determinants of Modern Paediatric Healthcare Seeking in Rural Côte d’Ivoire

**DOI:** 10.3389/ijph.2021.1604451

**Published:** 2022-01-31

**Authors:** Siaka Koné, Günther Fink, Nicole Probst-Hensch, Clémence Essé, Jürg Utzinger, Eliézer K. N’Goran, Marcel Tanner, Fabienne N. Jaeger

**Affiliations:** ^1^ Centre Suisse de Recherches Scientifiques en Côte d’Ivoire, Abidjan, Côte d’Ivoire; ^2^ Swiss Tropical and Public Health Institute, Allschwil, Switzerland; ^3^ University of Basel, Basel, Switzerland; ^4^ Institut d’Ethnosociologie, Université Félix Houphouët-Boigny, Abidjan, Côte d’Ivoire; ^5^ Unité de Formation et de Recherche Biosciences, Université Félix Houphouët-Boigny, Abidjan, Côte d’Ivoire

**Keywords:** determinants, child health, modern healthcare, Côte d’Ivoire, health and demographic surveillance system, mortality, Taabo

## Abstract

**Objectives:** To determine factors that influence healthcare seeking among children with fatal and non-fatal health problems.

**Methods:** Last disease episodes of surviving children and fatal outcomes of children under 5 years of age were investigated by means of an adapted social autopsy questionnaire administered to main caregivers. Descriptive analysis and logistic models were employed to identify key determinants of modern healthcare use.

**Results:** Overall, 736 non-fatal and 82 fatal cases were assessed. Modern healthcare was sought for 63.9% of non-fatal and 76.8% of fatal cases, respectively. In non-fatal cases, young age, caregiver being a parent, secondary or higher education, living <5 km from a health facility, and certain clinical signs (i.e., fever, severe vomiting, inability to drink, convulsion, and inability to play) were positively associated with modern healthcare seeking. In fatal cases, only signs of lower respiratory disease were positively associated with modern healthcare seeking. A lack of awareness regarding clinical danger signs was identified in both groups.

**Conclusion:** Interventions promoting prompt healthcare seeking and the recognition of danger signs may help improve treatment seeking in rural settings of Côte d’Ivoire and can potentially help further reduce under-five mortality.

## Introduction

Despite considerable progress made over the past two decades, under-five mortality remains high in many low- and middle-income countries (LMICs). Although countries have committed to providing equitable access to quality care for their citizens within the 2030 Agenda for Sustainable Development (2030 Agenda, in short), this objective remains a major challenge [1], particularly in settings characterized by persistent socioeconomic disparities in health and access to healthcare services [2–4]. To reach the ambitious mortality reduction goals set in the 2030 Agenda, improvements at the health system levels will likely have to go beyond basic provision of medical care [5–7].

An estimated 50% of the modifiable determinants of health have been attributed to community specific factors, which often determine an individual’s willingness and ability to access healthcare services [1, 6]. To deepen the understanding of the biomedical and social factors that influence disease progression, particularly in potentially fatal cases, social autopsy using a pathway analysis approach has been introduced in some countries [8–10]. Different from (complementary) verbal autopsies focusing on biomedical causes [11], social autopsies directly aim at determining factors contributing to health outcomes at the household, community, and health systems level. An improved understanding of these factors at different levels might aid prevention of morbidity and mortality, which seems particularly important in the context of infectious diseases (e.g., malaria), where timely provision of quality care is essential for patient survival.

In this study, we draw on rich social autopsy data obtained from the Taabo health and demographic surveillance system (HDSS), located in the south-central part of Côte d’Ivoire to identify key determinants of modern healthcare seeking for paediatric illness.

## Methods

### Ethics Statement

This study received ethical approval from Côte d’Ivoire (reference no. 172/MESRS/DGRSIT/tm) and Switzerland (reference no. EKBB: 263/13). Written informed consent was obtained from parents or legal guardians. Participants could withdraw from the study anytime without further obligations.

### Study Setting

The study was conducted in the Taabo HDSS. Established in late 2008 with an initial population of 37,792 [12, 13], by the end of 2018, the population in the Taabo HDSS had grown to approximately 48,000. The Taabo HDSS is located in a transition zone between tropical rain forest in the South and the savanna in the North, in a primarily rural area. There are 13 villages, over 100 hamlets, and one small town (i.e., Taabo-Cité). The health system is composed of a small hospital in Taabo-Cité and 10 primary healthcare centres located in the main villages. In the hamlets, no health facilities are available but community health workers (CHWs) provide basic health advice, guidance and services. According to verbal autopsy data collected over 3 years (from 2009 to 2011), 85% of child deaths were due to infectious diseases, with most deaths attributed to malaria and respiratory tract infections [13, 14].

### Study Design and Data Collection

We designed a prospective study with two complementary samples; 1) a sample of fatal episodes; and 2) a sample of non-fatal morbidity episodes. We defined fatal cases as children aged 1–59 months, who died in the Taabo HDSS within a 1-year period from January 2017 to January 2018. Child deaths were identified using a standard key informant system as part of regular demographic data collection rounds within the Taabo HDSS.

Non-fatal cases were randomly selected in the main villages from households inhabited by at least one child under the age of 5 years surveyed between January 2017 and January 2018. Overall, there were 780 household visits. During the visits, all illness episodes (not resulting in death) among children under the age of 5 years in the preceding 30 days were recorded. Whenever multiple episodes of illness occurred within the past 30 days, only information on the most recent illness past episode was collected. Of note, both for fatal and non-fatal cases, the study excluded newborns (<29 days of age), as to explicitly focus on the post-natal period.

### Social Autopsy

Social autopsy consisted of a face-to-face interview with the primary caregivers, usually the mother of the child, 2–8 weeks after the child’s death. The questionnaire was inspired by the Child Health Epidemiology Reference Group (CHERG) and the International Network for the continuous Demographic Evaluation for Populations and Their Health (INDEPTH) social autopsy questionnaires [9]. After initial questions on household characteristics, child survival, signs and symptoms observed, and disease recognition, questions explored the healthcare seeking behaviour (i.e., decision-making, choice and source of different home-based treatments, traditional and modern medicine providers, potential access barriers including accessibility, acceptability, availability, affordability, adequacy, and cultural factors). Presenting signs and symptoms were investigated with a special focus on those that 1) give information on the general state of the child; 2) indicate potentially severe disease; and 3) likely warrant consultation of a modern healthcare facility.

The questionnaire used for non-fatal cases was somewhat shorter, but was designed to cover the main aspects of the social autopsy form. The Open Data Kit (ODK) was used to build questionnaires in the national language French. Tablets were used for data collection by trained senior staff. ODK data as well as pictures of signed consent forms were uploaded and stored on a secure server.

### Quantitative Variables

The primary outcome of interest was a binary variable for modern healthcare seeking for a given illness episode. All caregivers who answered “yes” to the question “Have you consulted a provider of modern medicine?” were considered to have used modern medicine. Modern healthcare providers included hospital, public, or private health centres. Explanatory variables included 1) socio-demographic characteristics of the concerned child (i.e., age, sex, twin status, and relationship with the caregiver); 2) household and caregivers’ characteristics (socioeconomic status, distance to nearest health facility, educational attainment, age, etc.); and 3) signs and symptoms observed by the caregivers. Socioeconomic status was determined using a household-based asset approach using principal component analysis (PCA) with stratification into wealth quintiles [15]. We relied on household and health centres’ geographical coordinates, to estimate the distance from child’s place of residence to the nearest health facility by means of Statageodist package [16].

### Statistical Analysis

All statistical analyses were performed in Stata version 15.0 (StataCorp; College Station, TX, United States) [17]. Data records from caregivers with complete information on disease-related morbidity were considered for analysis. Multivariable standard logistic regression models were estimated to identify associations between modern healthcare seeking and a range of child, household, and community characteristics. Logistic regression results were presented as marginal effects (dy/dx), including 95% confidence interval (CI). Differences and relationships with a *p*-value below 0.05 were considered statistically significant.

## Results

### Study Sample

Overall, 754 children with recent non-fatal episodes and 104 deaths of children aged 1–59 months were identified through household visits in the Taabo HDSS over a 1-year period. Among the non-fatal cases, 736 had complete data records, and hence, were considered as final study sample (Supplementary Figure S1).

Among the fatal cases, 22 were excluded due to incomplete information, resulting in a final analytical sample of 82 fatal cases (Supplementary Figure S2).

### Caregivers, Child Socio-Demographic Characteristics, and Modern Treatment Seeking During Child’s Illness

Table 1 summarizes socio-demographic characteristics of caregivers and children, stratified by whether or not children were seeking modern care, both for fatal and non-fatal cases. Of the 736 children in the non-fatal sample, there were more boys (n = 412) than girls (n = 324). Among the 82 fatal cases, there were 43 boys and 39 girls. On average, children in the fatal cases group were younger (mean age: 14.9 vs. 29.8 months in the non-fatal group). While in the non-fatal cases 12.4% of children were younger than 1 year, this age group accounted for 47.6% among fatal cases. More than 85% of caregivers were 20 years and above.

**TABLE 1:** Socio-demographic characteristics, stratified by modern healthcare seeking for fatal and non-fatal cases. Social Autopsy Project, Côte d’Ivoire, 2017.

	Non-fatal cases			Fatal cases		
	N (%)	Modern healthcare seeking	No modern healthcare seeking	N (%)	Modern healthcare seeking	No modern healthcare seeking
Full sample	736 (100)	470 (63.9)	266 (36.1)	82 (100)	62 (75.6)	20 (24.4)
Child sex						
Male	412 (56.0)	272 (66.0)	140 (34.0)	43 (52.4)	28 (65.1)	15 (34.9)
Female	324 (44.0)	198 (61.1)	126 (38.9)	39 (47.6)	34 (87.2)	5 (12.8)
Child age (months)						
1–11	91 (12.4)	65 (71.4)	26 (28.6)	39 (47.6)	27 (69.2)	12 (30.8)
12–23	176 (23.9)	136 (77.3)	40 (22.7)	25 (30.5)	22 (88.0)	3 (12.0)
24–35	178 (24.2)	106 (59.6)	72 (40.4)	10 (12.2)	5 (50.0)	5 (50.5)
35–47	153 (20.8)	81 (52.9)	72 (47.1)	7 (8.5)	7 (100)	-
48–59	138 (18.7)	82 (59.4)	56 (40.6)	1 (1.2)	1 (100)	-
Child twin status						
Twin birth	17 (2.3)	11 (64.7)	6 (35.3)	3 (3.7)	1 (33.3)	2 (66.7)
Single birth	719 (97.7)	459 (63.8)	260 (36.2)	79 (96.3)	61 (77.2)	18 (22.8)
Maternal age (years)						
15–19	96 (13.0)	63 (65.6)	33 (34.4)	9 (11.0)	3 (33.3)	6 (66.7)
20–34	414 (56.3)	267 (64.5)	147 (35.5)	56 (68.3)	46 (82.1)	10 (17.8)
≥35	226 (30.7)	140 (62.0)	86 (38.0)	17 (20.7)	13 (76.5)	4 (23.5)
Average number of live birth*						
Number of live birth	3.7	3.6	3.9	4.2	4.3	3.9
Previous child death						
Yes	179 (35.7)	100 (55.9)	79 (44.1)	27 (51.9)	22 (81.5)	5 (18.5)
No	322 (64.3)	211 (65.5)	111 (34.5)	25 (48.1)	21 (84.0)	4 (16.0)
NA **	235 (.)	159 (.)	76 (.)	30 (.)	22 (.)	8 (.)
Child relationship with the caregiver						
Biological parents	403 (54.8)	263 (65.3)	140 (34.7)	42 (51.2)	36 (85.7)	6 (14.3)
Grandparents	256 (34.8)	165 (64.4)	91 (35.6)	36 (43.9)	22 (61.1)	14 (38.9)
Other	77 (10.4)	42 (54.6)	35 (45.4)	4 (4.9)	4 (100)	-
Main caregiver education						
None	401 (54.5)	250 (62.3)	151 (37.7)	66 (80.5)	49 (74.2)	17 (25.8)
Primary	221 (30.0)	142 (64.2)	79 (35.8)	5 (6.1)	3 (60.0)	2 (40.0)
Secondary or higher	86 (11.7)	64 (74.4)	22 (25.6)	10 (12.2)	9 (90.0)	1 (10.0)
Coranic	28 (3.8)	14 (50.0)	14 (50.0)	1 (1.2)	1 (100)	-
Household’s socioeconomic status						
Most poor	148 (20.1)	89 (60.1)	59 (39.9)	17 (20.7)	14 (82.3)	3 (17.7)
Less poor	148 (20.1)	90 (60.8)	58 (39.2)	16 (19.5)	10 (62.5)	6 (37.5)
Middle	147 (20.0)	97 (66.0)	50 (34.0)	17 (20.7)	10 (58.8)	7 (41.2)
Rich	152 (20.6)	106 (69.7)	46 (30.3)	18 (22.0)	16 (88.9)	2 (11.1)
Most rich	141 (19.2)	88 (62.4)	53 (37.6)	14 (17.1)	12 (85.7)	2 (14.3)
Household distance to nearest health facility (km)						
<1	160 (21.7)	107 (66.9)	53 (33.1)	18 (21.9)	15 (83.3)	3 (16.7)
1–4.9	469 (63.7)	308 (65.7)	161 (34.3)	55 (67.1)	38 (69.1)	17 (30.9)
≥5	107 (14.5)	55 (51.4)	52 (48.6)	9 (11.0)	9 (100)	-

*If mother is the main caregiver.

**No previous child or mother not main caregiver.

Among both non-fatal and fatal cases, more than 80% of children lived in households headed by at least one biological parent or grandparent. In general, the children were taken care of by either parents (fatal cases: 51.2% vs. non-fatal cases: 54.8%) or grandparents (fatal cases: 43.9% vs. non-fatal cases: 34.8%). Grandparents of non-survivors were less likely to have sought care at health services compared to parents or other caregivers (fatal cases grandparents: 61.1% vs. fatal cases biological parents: 85.7% or fatal cases other parents: 100%). In surviving children, care was less frequently sought for children not being a direct offspring.

When the main caregiver was the biological mother of the child, she was also asked about the number of live births and previous experiences of child loss. On average, mothers of fatal cases had given birth more often (fatal cases: 4.2 vs. non-fatal cases: 3.7 live births) and had experienced a previous loss of a child more frequently than mothers of non-fatal cases (44.3% vs. 30.1%). Mothers who had already lost a child were overall less likely to use modern healthcare than those who had not experienced a loss (fatal cases: 81.5% vs. non-fatal cases: 55.9%).

More than half of the main caregivers never attended school (54.5% in non-fatal cases sample vs. 80.5% in the fatal case sample). More than 80% of the children lived in households within 5 km of modern healthcare services (85.4% in non-fatal cases vs. 89.0% in fatal cases).

### Description of Disease Symptoms

Table 2 summarizes children’s general health state and signs and symptoms of diseases, as observed by caregivers, stratified by healthcare seeking for fatal and non-fatal cases, respectively. Modern care was sought for 63.9% and 75.6% of the children with non-fatal and fatal health problems, respectively. In surviving children, fever was the most commonly recognized symptom concerning nearly all investigated cases (95.4%). Among fatal cases, the reported fever prevalence was 72.0%. Signs of respiratory disease were reported in nearly 60% of non-fatal cases, of which approximately 10% showed signs of lower respiratory tract involvement. Lower respiratory tract involvement concerned fatal cases more frequently than non-fatal cases (15.8% vs. 5.6%). Gastrointestinal symptoms as well as symptoms of dehydration were common in both groups, with extreme thirst, more frequently observed by caregivers in the non-fatal group and severe vomiting and the inability to drink both nearly twice as frequent in the fatal group. Convulsions were five times more common in the fatal group (fatal cases: 23.2% vs. non-fatal cases: 4.8%).

**TABLE 2:** Description of disease symptoms by modern healthcare seeking for fatal and non-fatal cases. Social Autopsy Project, Côte d'Ivoire, 2017.

	Non-fatal cases			Fatal cases		
	Full sample	Modern healthcare seeking	No modern healthcare seeking	Full sample	Modern healthcare seeking	No modern healthcare seeking
	N = 736 (100%)	N = 470 (63.9%)	N = 266 (36.1%)	N = 82 (100%)	N = 62 (75.6%)	N = 20 (24.4%)
Fever						
Fever	702 (95.4)	459 (65.4)	243 (34.6)	59 (72.0)	49 (83.1)	10 (16.9)
Respiratory signs	438 (59.5)	264 (60.3)	174 (39.7)	18 (22.0)	13 (72.2)	5 (27.8)
Low respiratory	41 (5.6)	30 (73.2)	11 (26.8)	13 (15.8)	12 (92.3)	1 (7.7)
Diarrhoea	294 (40.0)	200 (68.0)	94 (32.0)	26 (31.7)	23 (88.5)	3 (11.5)
Severe diarrhoea^a^	210 (28.5)	149 (71.0)	61 (29.0)	23 (28.1)	20 (87.0)	3 (13.0)
Other diarrhoea	87 (11.8)	52 (59.8)	35 (40.2)	4 (4.9)	4 (100)	-
Vomiting	282 (38.3)	203 (72.0)	79 (28.0)	28 (34.1)	26 (92.9)	2 (7.1)
Severe vomiting^b^	74 (10.1)	62 (83.8)	12 (16.2)	15 (18.3)	14 (93.3)	1 (6.7)
Other vomiting	208 (28.3)	141 (67.8)	67 (32.2)	13 (15.8)	12 (92.3)	1 (7.7)
Risk of dehydration	418 (56.8)	295 (70.6)	123 (29.4)	44 (53.7)	37 (84.1)	7 (15.9)
Extremely thirsty	282 (38.3)	193 (68.44)	89 (31.6)	16 (19.5)	14 (87.5)	2 (12.5)
Unable to drink	66 (9.0)	57 (86.4)	9 (13.6)	18 (22.0)	13 (72.2)	5 (27.8)
Neurological signs	455 (61.8)	321 (70.6)	134 (29.4)	78 (95.1)	61 (78.2)	17 (21.8)
Convulsion	35 (4.8)	34 (97.1)	1 (2.9)	19 (23.2)	15 (79.0)	4 (21.0)
Stiff neck	7 (0.9)	7 (100)	-	2 (2.4)	2 (100)	-
Reduced or loss of consciousness	450 (61.1)	317 (70.4)	133 (29.6)	76 (92.7)	63 (82.9)	13 (17.1)
Urinary change	408 (55.4)	289 (70.8)	119 (29.2)	7 (8.5)	6 (85.7)	1 (14.3)
Bad smell	249 (33.8)	161 (64.7)	88 (35.3)	4 (4.9)	4 (100)	-
Dark urine	321 (43.6)	231 (72.0)	90 (28.0)	4 (4.9)	3 (75.0)	1 (25.0)
Pollakisuria or/and dysuria	9 (1.2)	8 (88.9)	1 (11.1)	-	-	-
Less urine	7 (0.9)	6 (85.7)	1 (14.3)	1 (1.2)	1 (100)	-
Blood in urine	3 (0.4)	3 (100)	-	-	-	-
Eyes	262 (35.6)	195 (74.4)	67 (25.6)	22 (26.8)	18 (81.8)	4 (18.2)
Very swollen	5 (0.7)	1 (20.0)	4 (80.0)	-	-	-
Red or discharging	7 (0.9)	6 (85.7)	1 (14.3)	-	-	-
Yellow	183 (24.9)	131 (71.6)	52 (28.4)	8 (9.8)	8 (100)	-
Other	67 (9.1)	57 (85.1)	10 (14.9)	14 (17.1)	10 (71.4)	4 (28.6)
Rash						
Rash	53 (7.2)	38 (71.7)	15 (28.3)	6 (7.3)	5 (83.3)	1 (16.7)
Strong pain	80 (10.8)	48 (60.0)	32 (40.0)	4 (4.9)	4 (100)	-
Stomach	32 (4.3)	19 (59.4)	13 (40.6)	3 (3.7)	3 (100)	-
Head	23 (3.1)	13 (56.5)	10 (43.5)	-	-	-
Thorax	9 (1.2)	7 (77.8)	2 (22.2)	1 (1.2)	1 (100)	-
Other	16 (2.2)	9 (56.3)	7 (43.7)	-	-	-
Change of colour of skin	232 (31.5)	167 (72.0)	65 (28.0)	53 (64.6)	44 (83.0)	9 (17.0)
Pallor or cyanoses	224 (30.4)	161 (71.9)	63 (28.1)	52 (63.4)	43 (82.7)	9 (17.3)
Jaundice	15 (2.0)	13 (86.7)	2 (13.3)	2 (2.4)	2 (100)	-
General state at disease recognition						
Vigilance	359 (48.8)	247 (68.8)	112 (31.2)	75 (91.5)	62 (82.7)	13 (17.3)
Alert	336 (45.6)	197 (58.6)	139 (41.4)	6 (7.3)	3 (50.0)	3 (50.0)
Reduced	356 (48.4)	244 (68.5)	112 (31.5)	75 (91.5)	62 (82.7)	13 (17.3)
Unconscious	3 (0.4)	3 (100)	-	-	-	-
Doesn’t know	41 (5.6)	26 (63.4)	15 (36.6)	-	-	-
Sudden death	-	-	-	1 (1.2)	-	1 (100)
Activity (play)	605 (82.2)	413 (68.3)	192 (31.7)	74 (90.2)	61 (82.4)	13 (17.6)
Active	118 (16.0)	45 (38.1)	73 (61.1)	7 (8.5)	4 (57.1)	3 (42.9)
Reduced activity	456 (62.0)	291 (63.8)	165 (36.2)	40 (48.8)	36 (90.0)	4 (10.0)
None	149 (20.2)	122 (81.9)	27 (18.1)	34 (41.5)	25 (73.5)	9 (26.5)
Doesn’t know	13 (1.8)	12 (92.3)	1 (7.7)	-	-	-
Sudden death	-	-	-	1 (1.22)	-	1 (100)
Food intake	532 (72.3)	359 (67.5)	173 (32.5)	73 (89.0)	62 (84.9)	11 (15.1)
Normal food intake	203 (27.6)	110 (54.2)	93 (45.8)	8 (9.8)	3 (37.5)	5 (62.5)
Less good	428 (58.2)	283 (66.1)	145 (33.9)	54 (65.8)	50 (92.6)	4 (7.4)
None	104 (14.1)	76 (73.1)	28 (26.9)	19 (23.2)	12 (63.2)	7 (36.8)
Doesn’t know	1 (0.1)	1 (100)	-	-	-	-
Sudden death	-	-	-	1 (1.2)	-	1 (100)
General state when worst^c^						
Vigilance	447 (60.7)	314 (70.3)	133 (29.7)	-	-	-
Alert	272 (37.0)	142 (52.2)	130 (47.8)	-	-	-
Reduced	431 (58.5)	299 (69.4)	132 (30.6)	-	-	-
Unconscious	16 (2.2)	15 (93.7)	1 (6.3)	-	-	-
Doesn’t know	17 (2.3)	14 (82.4)	3 (17.6)	-	-	-
Activity (play)	665 (90.3)	447 (67.2)	218 (32.8)	-	-	-
Active	71 (9.6)	23 (32.4)	48 (67.6)	-	-	-
Reduced activity	412 (56.0)	262 (63.6)	150 (36.4)	-	-	-
None	253 (34.4)	185 (73.1)	68 (26.9)	-	-	-
Food intake	619 (84.1)	413 (66.7)	206 (33.3)	-	-	-
Normal food intake	115 (15.6)	56 (48.7)	59 (51.3)	-	-	-
Less good	431 (58.6)	283 (65.7)	148 (34.3)	-	-	-
None	188 (25.5)	130 (69.2)	58 (30.8)	-	-	-
Doesn’t know	2 (0.3)	1 (50.0)	1 (50.0)	-	-	-

a Severe diarrhoea: more than 4–5 liquid stools/day or at least 3x/day for 48 h.

b Severe vomiting: frequent vomiting for more than 24 h or vomiting everything a child tries to drink over a period of several hours.

c General state when worst: child general state when the health problem was most severe.

Reduced or loss of consciousness were frequently reported both among surviving children (61.1%) and in the fatal disease group (92.7%). Urinary changes were more commonly observed in the non-fatal group, and pallor or cyanoses were observed twice as often in the fatal (63.4%) than in the non-fatal group (30.4%). No child died due to an accident.

### Factors Associated With Modern Healthcare Seeking

Table 3 presents results from the fully adjusted model, summarizing the associations between modern healthcare seeking and the characteristics of the child, household, and main caregiver. In non-fatal cases, children aged 1–11 months were 11%-points (95% CI: −2%-points to 24%-points; *p*-value <0.1) and children aged 12–23 months were 16%-points (95% CI: 6%-points to 27%-points; *p*-value <0.01) more likely to benefit from modern care than children aged 48–59 months. Caregivers who never attended school and those who attained primary and coranic school levels were 11%-points (95% CI: −22%-points to 1%-point; *p*-value <0.05), and caregivers only attending coranic schools were 27%-points (95% CI: −47%-point to −7%-point; *p*-value <0.01) less likely to utilize modern medicine treatment than more highly educated caregivers. Households living less than 1 km from the nearest health facility, had a 14%-points (95% CI: 1%-point to 26%-point; *p*-value <0.05) and those households living 1–4 km away a 12%-points (95% CI: 2%-points to 23%-points, *p*-value <0.05) higher propensity to attend a modern healthcare provider than those living at least 5 km away.

**TABLE 3:** Association between socio-demographic factors and modern healthcare seeking. Social Autopsy Project, Côte d'Ivoire, 2017.

	Non-fatal cases	Fatal cases
	dy/dx (95% CI)	dy/dx (95% CI)
Child sex: Reference: male		
Female	−0.05 (−0.12, 0.02)	0.06 (−0.12, 0.25)
Child age (months): Reference: 48–59 months		
1–11	0.11* (−0.02, 0.24)	0.07 (−0.56, 0.70)
12–23	0.16*** (0.06, 0.27)	0.13 (−0.47, 0.73)
24–35	−0.02 (−0.13, 0.09)	−0.22 (−0.91, 0.48)
35–47	−0.09 (−0.21, 0.03)	0.04 (−0.66, 0.75)
Child twin status: Reference: twin		
Single	−0.01 (−0.24, 0.22)	0.33* (−0.00, 0.67)
Child relationship with the main caregiver: Reference: biological parent		
Grandparent	0.15** (0.02, 0.27)	−0.13 (−0.42, 0.15)
Other	0.01 (−0.18, 0.20)	0.01 (−0.56, 0.59)
Maternal age (in years): Reference: ≥35 years		
15–19	0.01 (−0.10, 0.13)	−0.41** (−0.77, −0.06)
20–34	0.01 (−0.07, 0.09)	0.12 (−0.13, 0.36)
Main care giver education: Reference: secondary or higher		
None	−0.11** (−0.22, −0.01)	−0.02 (−0.28, 0.24)
Primary	−0.11* (−0.22, 0.00)	−0.20 (−0.61, 0.22)
Coranic	−0.27*** (−0.47, −0.07)	−0.05 (−0.67, 0.57)
Household’s socioeconomic status: Reference: most poor		
Poor	0.00 (−0.10, 0.11)	0.10 (−0.22, 0.42)
Middle	0.03 (−0.08, 0.14)	0.08 (−0.23, 0.38)
Rich	0.08 (−0.03, 0.19)	0.26 (−0.08, 0.60)
Most rich	−0.03 (−0.14, 0.09)	0.02 (−0.30, 0.35)
Household distance to nearest health facility: Reference ≥5 km		
<1 km	0.14** (0.01, 0.26)	−0.21 (−0.54, 0.12)
1–4.9 km	0.12** (0.02, 0.23)	−0.19 (−0.45, 0.07)
Observations	736	82

***p < 0.01.

**p < 0.05.

*p < 0.1.

Coefficients displayed are dy/dx = marginal effect is a change in the probability that Y = 1 with a specific change in X with 95% confidence intervals (CIs) in parentheses; adjusted models control for household, child, and caregiver’s characteristics.

Among fatal cases, caregivers or mothers aged 15–19 years were 41%-points less likely to seek modern healthcare than women aged 35 and older (95% CI: −77%-point to −6%-point; *p*-value <0.05).

### Observed Symptoms Associated With Modern Healthcare Seeking

Table 4 shows the predicted effects of disease signs and symptoms on modern healthcare seeking from alternative logistic models.

**TABLE 4:** Association between disease symptoms and modern healthcare seeking. Social Autopsy Project, Côte d’Ivoire, 2017.

Variable	Non-fatal cases			Fatal cases	
	Model 1	Model 2	Model 3	Model 4	Model 5
	dy/dx (95% CI)	dy/dx (95% CI)	dy/dx (95% CI)	dy/dx (95% CI)	dy/dx (95% CI)
Disease signs and symptoms					
Fever	0.25*** (0.09, 0.41)	0.13 (−0.03, 0.29)	0.20** (0.03, 0.37)	0.26** (0.06, 0.46)	0.18 (−0.08, 0.43)
Lower respiratory	−0.03 (−0.20, 0.15)	0.06 (−0.11, 0.23)	−0.02 (−0.19, 0.14)	0.38*** (0.12, 0.65)	0.42*** (0.14, 0.70)
Severe diarrhoea	0.03 (−0.05, 0.11)	0.03 (−0.06, 0.11)	0.00 (−0.08, 0.09)	0.18 (−0.09, 0.44)	0.14 (−0.15, 0.43)
Severe vomiting	0.19*** (0.06, 0.33)	0.13** (0.00, 0.27)	0.16** (0.03, 0.30)	0.06 (−0.26, 0.37)	0.21 (−0.09, 0.50)
Extremely thirsty	0.06 (−0.02, 0.14)	0.05 (−0.03, 0.12)	0.06 (−0.02, 0.13)	0.26 (−0.15, 0.68)	0.21 (−0.16, 0.58)
Inability to drink	0.19** (0.02, 0.36)	0.19** (0.03, 0.35)	0.19** (0.03, 0.35)	0.04 (−0.18, 0.26)	0.00 (−0.26, 0.26)
Convulsion	0.54** (0.12, 0.96)	0.53*** (0.13, 0.92)	0.52** (0.11, 0.93)	−0.10 (−0.35, 0.15)	−0.08 (−0.33, 0.17)
Bad-smelling urine	−0.08** (−0.16, −0.01)	−0.10*** (−0.18, −0.03)	−0.08** (−0.16, −0.00)	0.20 (−0.38, 0.78)	0.32 (−0.10, 0.73)
Dark urine	0.08** (0.01, 0.16)	0.09** (0.01, 0.17)	0.06 (−0.02, 0.14)	−0.65*** (−1.10, −0.19)	−0.60*** (−0.97, −0.23)
Less urine	0.33 (−0.12, 0.77)	0.34 (−0.08, 0.76)	0.31 (−0.13, 0.75)	0.15 (−0.33, 0.64)	−0.07 (−0.55, 0.42)
Yellow eyes	0.06 (−0.03, 0.15)	0.08* (−0.01, 0.16)	0.05 (−0.04, 0.14)	0.14 (−0.31, 0.60)	0.08 (−0.29, 0.45)
Other eye affections (swollen, discharging, other)	0.15** (0.01, 0.29)	0.15** (0.01, 0.29)	0.11 (−0.04, 0.25)	−0.19* (−0.39, 0.01)	−0.03 (−0.27, 0.21)
Skin rash	0.11 (−0.03, 0.26)	0.08 (−0.06, 0.22)	0.10 (−0.04, 0.24)	−0.09 (−0.42, 0.25)	−0.14 (−0.47, 0.20)
Strong pain	−0.07 (−0.18, 0.04)	−0.09 (−0.19, 0.02)	−0.11** (−0.22, −0.01)	0.26 (−0.44, 0.95)	0.12 (−0.37, 0.62)
Change of colour of skin	−0.01 (−0.10, 0.08)	−0.01 (−0.10, 0.08)	−0.00 (−0.09, 0.09)	0.11 (−0.12, 0.35)	0.06 (−0.20, 0.32)
General state at disease beginning					
Played less than usual		0.18*** (0.07, 0.29)			0.14 (−0.54, 0.81)
Did not play at all		0.50*** (0.34, 0.66)			−0.24 (−0.95, 0.48)
Ate less than usual		−0.14*** (−0.24, −0.04)			0.03 (−0.42, 0.47)
Did not eat at all		−0.27*** (−0.44, −0.10)			0.13 (−0.40, 0.66)
Awareness decreased/unconsciousness		0.07* (−0.01, 0.14)			0.09 (−0.88, 1.06)
General state at worst disease state					
Played less than usual			0.24*** (0.10, 0.39)		
Did not play at all			0.36*** (0.19, 0.53)		
Ate less than usual			−0.12* (−0.25, 0.02)		
Did not eat at all			−0.23*** (−0.39, −0.06)		
Awareness decreased/unconsciousness			0.08** (0.00, 0.16)		
Observations	736	736	736	82	82

*p < 0.1.

**p < 0.05.

***p < 0.01.

Coefficients displayed are dy/dx = marginal effect is a change in the probability that Y = 1 with a specific change in X with 95% confidence intervals (CIs) in parentheses. Model 1 and model 4 control for disease’s symptoms only; model 2 and model 5 control for disease’s symptoms and child’s general state at the beginning of the disease; model 3 controls for disease’s symptoms and child’s general state at worst disease state.

In the adjusted analysis of fatal cases (model 5), only signs of lower respiratory track involvement were positively associated with modern healthcare seeking (dy/dx = 0.42; 95% CI: 0.14–0.70; *p*-value <0.05). In contrast, dark urine was negatively associated with modern healthcare seeking (dy/dx = −0.60; 95% CI: −0.97 to −0.23; *p*-value <0.05). No associations between modern healthcare seeking and any other symptoms were observed.

In non-fatal cases, when considering only disease-related symptoms, fever (dy/dx = 0.25; 95% CI: 0.09–0.41; *p*-value <0.01), severe vomiting (dy/dx = 0.19; 95% CI: 0.06–0.33; *p*-value <0.01), inability to drink (dy/dx = 0.19; 95% CI: 0.02–0.36; *p*-value <0.05), convulsions (dy/dx = 0.54; 95% CI: 0.12–0.96; *p*-value <0.05), dark urine (dy/dx = 0.08; 95% CI: 0.01–0.16; *p*-value <0.05), and eye infection (dy/dx = 0.15; 95% CI: 0.01–0.29; *p*-value <0.05) were positively associated with modern healthcare seeking. Bad-smelling urine was negatively associated with treatment seeking (dy/dx = −0.08; 95% CI: −0.16 to −0.01; *p*-value <0.05).

Similar marginal effects and trends in symptoms were observed when the general state of the child’s health at the beginning of the disease and during the disease’s severity was taken into account. Children who were less able to play than usual at the beginning of the disease episode were associated with an 18%-point (95% CI: 7%-point to 29%-point; *p*-value <0.01) increase in modern treatment seeking and those who were not able to play at all a 50%-point (95% CI: 34%-point to 66%-point; *p*-value <0.01) increase. Unconsciousness was associated with a 7%-point increased in treatment seeking (95% CI: −1%-point to 14%-point; *p*-value <0.1). Similar results were obtained when disease severity symptoms at the point when the disease was most severe were analyzed (model 3). In contrast, modern treatment seeking was not taken up when the child ate less than usual or could not eat at all.

With regard to symptoms in fatal cases, fever (dy/dx = 0.26; 95% CI: 0.06–0.46; *p*-value <0.05) and lower respiration tract infections (dy/dx = 0.38; 95% CI: 0.12–0.65; *p*-value <0.01) were positively associated with modern healthcare seeking (model 4). The change of urine color was negatively associated with modern treatment seeking.

### Reasons for Not Seeking Modern Care

**FIGURE 1:**
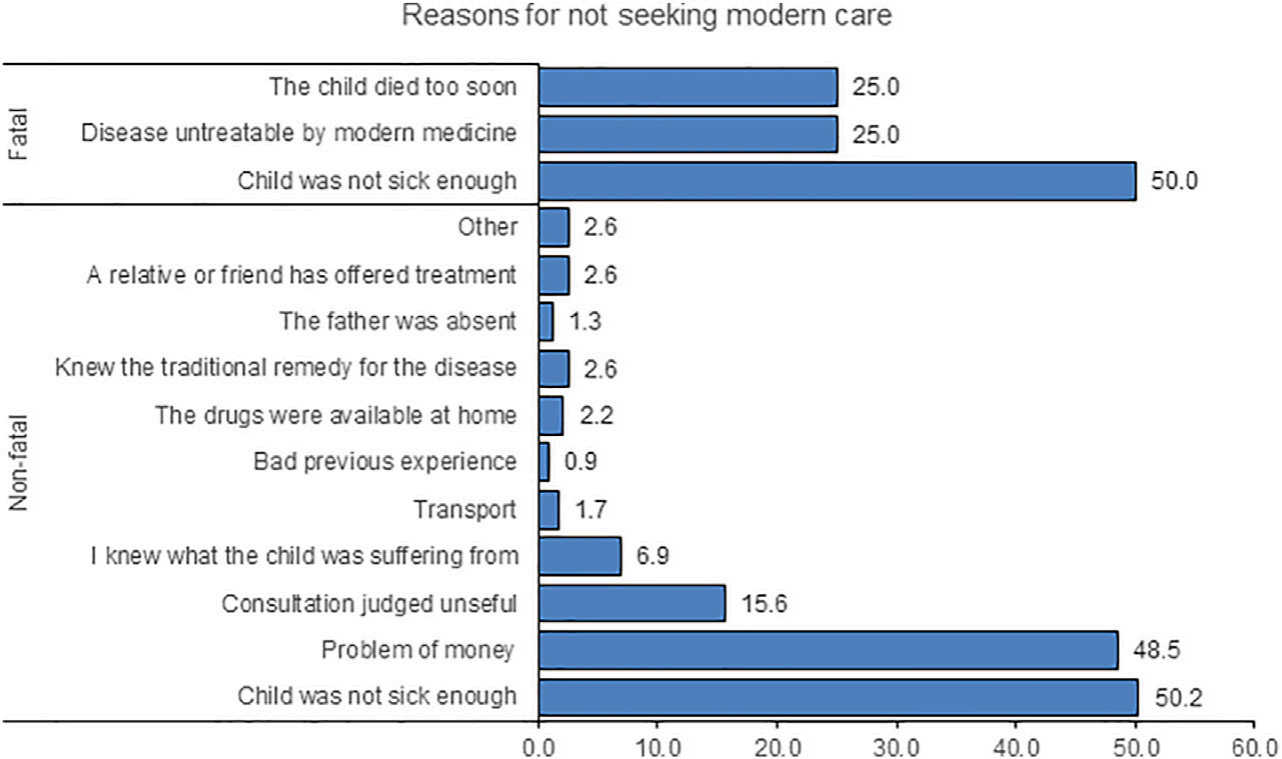
Reasons for not seeking modern healthcare, stratified by cases. Social Autopsy Project, Côte d’Ivoire, 2017.

Figure 1 shows the primary reasons caregivers stated for not seeking care. In non-fatal cases, among the 231 respondents, 50.2% felt that the child was not sick enough, 48.5% highlighted financial constraints, 15.6% did not consider consulting useful, while 6.9% indicated to know what the child was suffering from without consultation. Only 2.2% of caregivers said that they had used drugs already available at home instead of going to a health facility. Transportation difficulties, the absence of the child’s father, and unpleasant previous experience with modern health service were reported by 1.7%, 1.3%, and 0.9%, respectively.

In fatal cases, the main reason for not seeking care was that the child was not considered sick enough (50%). Other reasons were the perception that the disease was not treatable by modern medicine (25%) or the fact that the child died so soon after occurrence of symptoms (25%).

## Discussion

This study identified several determinants of modern healthcare seeking in a primarily rural setting in the south-central part of Côte d’Ivoire. Almost two-third (64%) of caregivers of children aged 1–59 months experiencing a non-fatal health condition sought care. This percentage is considerably higher than the 45.2% reported in a national study in 2016 [18]. Our estimates exclusively focus on the use of modern healthcare services. However, in the African context, care-seeking behaviours are complex and often characterized by a combination of modern and traditional medicine [19, 20], and sequential treatment seeking at different systems [21, 22]. Increased treatment-seeking propensity among younger children, among children living with their grandparents, and among caregivers with higher educational attainment appear well aligned with previous research [23–25].

Preceding studies have highlighted geographical distance and socioeconomic status [26–28] as key determinants for health services use. In our study, close proximity to the nearest healthcare facility was associated with increased health seeking for non-fatal cases. Though financial resources were mentioned as a factor for not accessing modern healthcare, we did not find any significant association across socioeconomic groups. This finding is explained by healthcare officially being offered free of charge in Côte d’Ivoire to pregnant women and children under the age of 5 years since 2012 [29]. In Burkina Faso, user fee removal was associated with the use of modern health services across all socioeconomic groups, but this was without adjustment for health needs and distance to health centres [30]. Other studies have shown the effect of wealth on health seeking behaviour, and hence, attributed delays to shortages of financial resources [31, 32]. More than three-quarters of fatal cases sought modern care prior to the death of the child. Caregivers aged <20 years were 41% less likely to bring their child to a modern healthcare provider than those aged ≥35 years, which is in line with previous research [23, 28, 33]. A likely explanation of this observation is that older mothers are more experienced. Compared to those caring for twins, caregivers caring for a single child were more likely to use modern healthcare, suggesting that increased care duties may interfere with timely treatment seeking.

Interestingly, mothers of the fatal cases were more likely to have already experienced the loss of a child. Both in surviving and non-surviving children, mothers with a previous loss were less likely to attend healthcare. Targeted interventions with a non-blaming, empathetic approach focusing on families who have already lost a child, may help increase treatment seeking among high-risk populations and prevent further deaths among young children.

Previous studies indicate the knowledge of danger signs and perceived severity of the illness to be associated with seeking modern healthcare [34, 35]. In our study, signs and symptoms most strongly associated with healthcare seeking were fever, severe vomiting, inability to drink, convulsion, or dark urine and factors inherent to a child’s general state (e.g., child playing less than usual, complete inactivity, decreased vigilance, or unconsciousness). Strikingly, while in surviving children all but one who presented with convulsions were brought in for modern care, the same was true in as many as 20% among one in five children with fatal disease outcomes, suggesting a lack of recognition of danger signs as a key barrier to timely treatment seeking with severe disease.

While higher rates of signs and symptoms typical for potentially severe disease are expected in fatal cases (e.g., signs of lower respiratory infection), a generally worse state at the time of disease onset may not just be due to more severe disease but also due to late recognition thereof. For non-fatal cases, not seeking external healthcare on the basis that the child was not sick enough, as revealed in the present study, suggests a non-recognition or an underestimation of the signs and symptoms expressed by the child and a lack of knowledge on diseases [28, 36]. Poor recognition may also hold for fever, the most frequently reported symptom in our study setting, which was only recognized in 72% of fatal cases, while in non-fatal cases fever was reported in 95%. The high rates of fever and results of the general state variables indicate that many surviving children may have had severe disease, and that pauci-symptomatic illnesses go unnoticed or are not classified as “a child being sick” by caregivers in the Taabo HDSS.

In our study, 36.1% and 24.4% of caregivers did not seek modern healthcare in non-fatal and fatal cases, respectively. Rather strikingly, in fatal cases, two thirds of the caregivers who did not seek modern healthcare were grandparents and teenage caregivers. Some of the caregivers (with fatal outcomes) appear to have shied away from seeking modern healthcare because they felt a consultation would not be useful, suggesting somewhat limited trust in the local health system. In some cases, a very poor general state (i.e., inactivity, loss of consciousness, and inability to drink) also appears to have resulted in caregivers considering the case lost (too late to seek care). Even though grandparents should have more experience, exposure to modern medicine may be more limited, while past experiences with death may induce beliefs that severe disease courses may be irreversible.

Local norms and beliefs regarding the aetiology of disease [37, 38] appear important. For example, a mother who lost a child in our study reported that the grandparents prevented a child being brought to a modern healthcare facility, as they considered the disease to be treated by prayer alone. In that sense, our results are consistent with several other studies, which have highlighted the importance of the caregivers’ skills and educational attainment in child health management [34, 36, 37].

In this study, we used data prospectively collected within a population of more than 45,000 continuously monitored in a well-defined geographical area. This has the advantage of identifying fatalities thoroughly and drawing on a representative sample. While other studies looking at determinants of healthcare seeking have focused on specific pathologies, signs and symptoms, or on fatal cases only, our study provides a more general analysis in both non-fatal and fatal cases of children aged 1–59 months. The fact that the data used relate to the last episode of illness may limit our conclusions as care seeking may vary from one episode to another. In order to gain as representative a picture as possible though, we ensured homogenous data collection over a 1-year period, thus also including seasonal variations. Furthermore, not every caregiver will adhere to the same definition of disease. While the two groups examined cannot be compared directly as the nature of diseases are likely to have been different with numerous illnesses with spontaneous resolution in the surviving group, the focus on signs and symptoms warranting consultation allows for a certain comparison of likelihood of action taken and importance attributed to them. As the responses collected from caregivers relate to the most recent event, a certain recall bias cannot be excluded, though episodes did not lay far back. Social desirability may have played a role though confidentiality was ensured and a very respectful, non-judgmental attitude was observed to try and minimize this issue.

### Conclusion

The findings presented here from a primarily rural part of Côte d’Ivoire suggest that an increasingly large proportion of caregivers seek modern care for their under 5-year-old children. Nonetheless, a quarter of fatal, and more than a third of non-fatal diseases, were not seen by modern healthcare providers. Increasing the perceived need and urgency of treatment for severe cases as well as improving the overall quality of care are of critical importance if further improvements reducing child mortality are to be achieved in LMICs.

## Supplementary Material

Supplementary Figure S1 Flow chart indicating non-fatal cases registered in 2017 in the Taabo health and demographic surveillance system in the south-central part of Côte d’Ivoire. Social Autopsy Project, Côte d’Ivoire, 2017.

Supplementary Figure S2 Flow chart indicating fatal cases registered in 2017 in the Taabo health and demographicsurveillance system in the south-central part of Côte d’Ivoire. Social Autopsy Project, Côte d’Ivoire, 2017.
