# Supplementary figures and images for: Determinants of Modern Paediatric Healthcare Seeking in Rural Côte d’Ivoire
Source: Int J Public Health. 2022 Jan 31;66:1604451. doi: 10.3389/ijph.2021.1604451 (PMC8842662; doi:10.3389/ijph.2021.1604451)

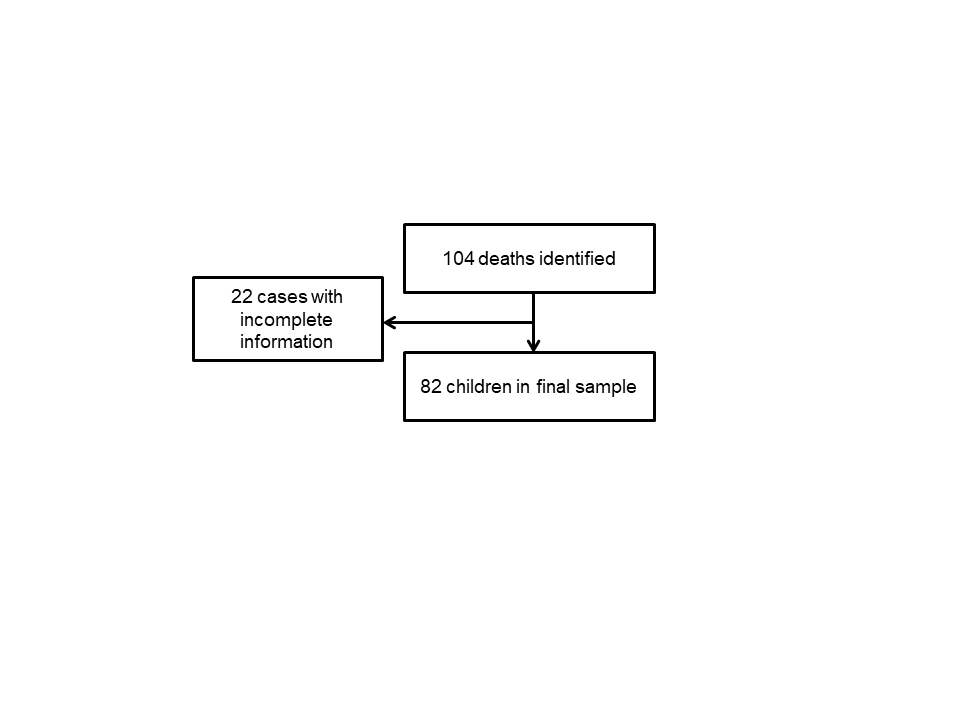

Supplement: Supplementary file 1 [file Image2.tif]

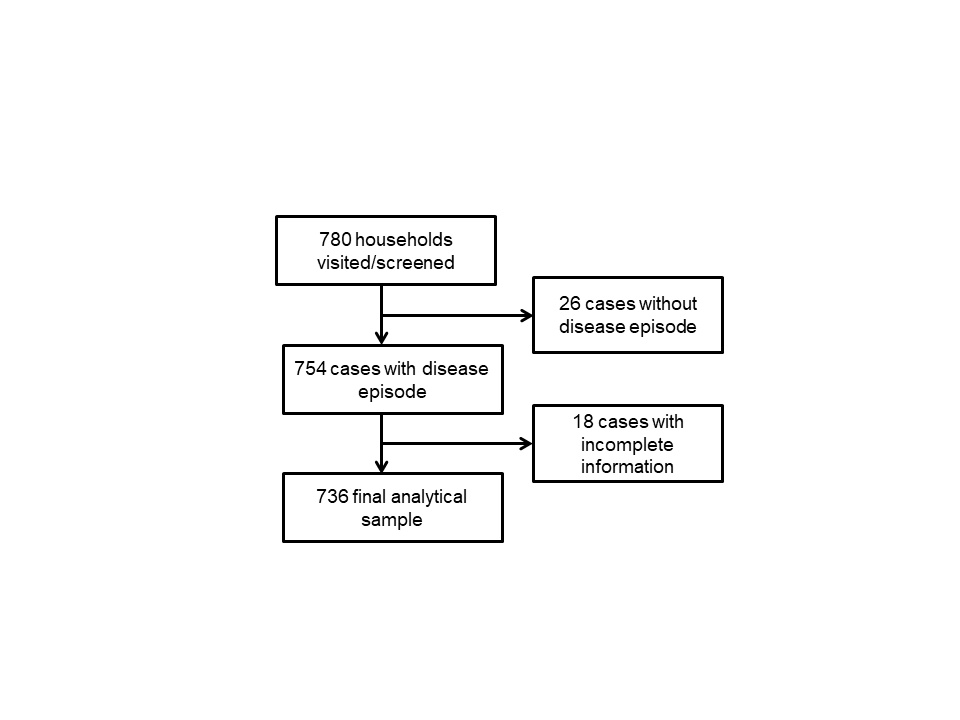

Supplement: Supplementary file 2 [file Image1.tif]
